# Interleukin-11-induced capillary leak syndrome in primary hepatic carcinoma patients with thrombocytopenia

**DOI:** 10.1186/1471-2407-11-204

**Published:** 2011-05-27

**Authors:** Wang Kai-Feng, Pan Hong-Ming, Lou Hai-Zhou, Shen Li-Rong, Zhu Xi-Yan

**Affiliations:** 1 Department of Oncology, Sir Run Run Shaw Hospital, Medical School, Zhejiang University, 3 Qingchun Dong Street, Hangzhou, China

## Abstract

**Background:**

Capillary leak syndrome (CLS) is a rare condition characterized by recurrent episodes of generalized edema and severe hypotension associated with hypoproteinemia. Interleukin-11 (IL-11) is a promising therapeutic agent for thrombocytopenia. A direct correlation between IL-11 and CLS has never been reported previously, particularly in patients with hepatic carcinoma.

**Case presentation:**

We describe two cases of CLS after IL-11 administration in two males with thrombocytopenia. Case 1 was a 46-year-old man with recurrence of hepatic carcinoma who was treated with IL-11 (3 mg per day). After four days of therapy, hypotension and hypoproteinemia were detected. The chest X-ray and B ultrasound of the abdomen showed pleural effusion and ascites. IL-11 was then discontinued, fluid resuscitation was performed, and fresh frozen plasma and packed red blood cells were transfused into this patient. The patient had recovered after 19 days of treatment.

Case 2 was a 66-year-old man who had undergone radiofrequency ablation (RFA) for hepatic carcinoma. He was treated with IL-11 (3 mg per day) for thrombocytopenia. After two days of therapy, this patient complained of dyspnea with bilateral edema of the hands. Laboratory values showed hypoproteinemia. IL-11 was stopped and human albumin was transfused at a rate of 10 g per day. On the 4^th ^day, fluid resuscitation was performed. The patient had recovered after treatment for two weeks.

**Conclusions:**

The detection of IL-11-induced CLS supports the hypothesis that CLS could be a severe side effect of IL-11 treatment in some patients. These two case reports also demonstrate that patients with hepatic carcinoma who experience this rare form of CLS after treatment with IL-11 seem to respond to a therapeutic regimen that involves hydroxyethyl starch, albumin, and diuretic therapy. Liver cancer patients might be more susceptible to CLS because of poor liver function and hypersplenia. In addition, bleeding after RFA might be a further inducer of CLS.

## Background

Capillary leak syndrome (CLS) is a rare clinical syndrome that was first described in 1960. In the vast majority of cases, it is characterized by acute episodes of generalized edema, hemoconcentration, and hypoproteinemia. The cause of CLS is unknown, and the condition is probably under recognized because of its nonspecific symptoms and signs and high mortality rate. Up to and including 1990, 25 cases of CLS had been analyzed in total worldwide [1]. In 2007, Matsumura et al. reported that approximately 57 cases had been identified in total, 12 of whom died of the disease during follow-up [2]. However, in the same year, Dhir et al. stated that there had been 75 additional reports of CLS since the review published in 1990 [3]. We have found 17 reports in the last 3 years; four of which occurred in infants and none in patients with liver cancer. Therefore, the number of reported cases of this syndrome now totals 117, and has shown an increasing trend since this syndrome was first recognized. In contrast to the poor survival noted previously, the prognosis of this syndrome now seems to be fair, with a 5-year survival rate of 70%. Further understanding of this enigmatic syndrome is needed.

Many drugs are known to induce CLS, including interleukin-2 (IL-2) [4], gemcitabine [5], doxorubicin [6], granulocyte colony stimulating factor (G-CSF) [7], and interferon [8]. Hsiao et al. described the first case of CLS induced by molecular target medicine in 2010 [9]. Miscellaneous conditions such as carbon monoxide poisoning [10], postpartum state [11], and pustular psoriasis [12] have been also associated with CLS.

To our knowledge a correlation between CLS and IL-11 in patients with liver cancer has never been reported. The identification of IL-11-induced CLS is quite important, because it supports the hypothesis that IL-11 is responsible for the pathogenesis of IL-11-related acute hypotension. Given that no effective treatment for CLS has been found so far, CLS can lead to death if the blood pressure is not increased during the initial capillary leak phase. Usually CLS responds successfully to hydroxyethyl starch, albumin, and diuretics.

## Case Presentation

### Case 1

On May 27^th ^2008, a 46-year-old man was admitted to our hospital with hypersplenia after radiofrequency ablation (RFA) for liver cancer. On the day of admission, the blood pressure of the patient was 122/76 mmHg, heart rate was 72 beats per minute (bpm), respiratory rate was 20 per minute, and oral body temperature was 36.9°C. The chest computed tomography (CT) scan was normal. Laboratory values were as follows: leukocytes count 3.0 × 10^9^/L; hemoglobin 12 g/dL; hematocrit 35.8%; platelet count 48 × 10^9^/L; albumin 3.2 g/dL; aspartate aminotransferase (AST) 82 IU/L (normal 5-35 IU/L); alanine aminotransferase (ALT) 51.1 IU/L (normal 5-45 IU/L). Therefore, IL-11 was administered subcutaneously at a dose of 3 mg per day because of thrombocytopenia.

**Figure 1:**
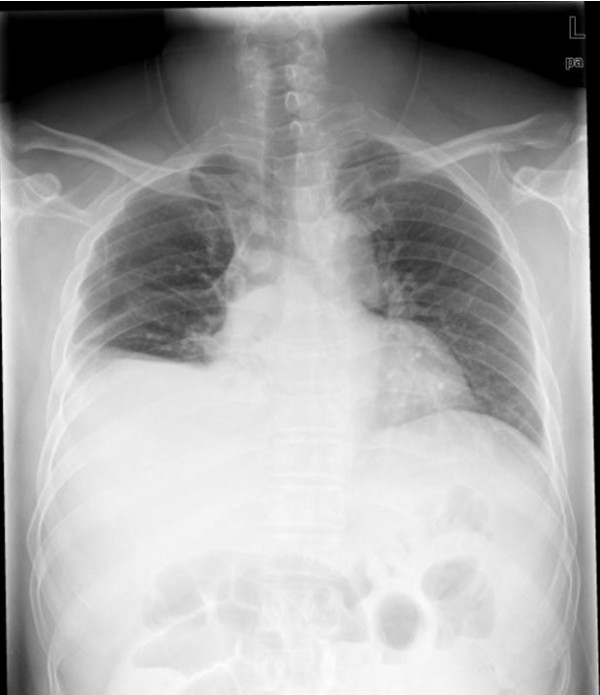
**The right diaphragm and costophrenic angle were obscured**. There was moderate right pleural effusion in case 1 after treatment with IL-11.

**Figure 2:**
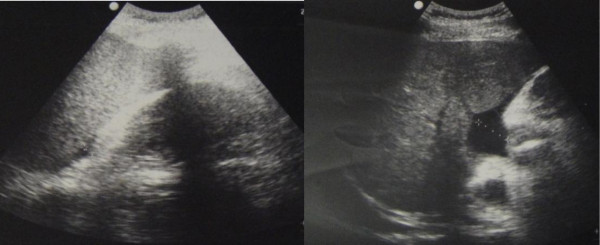
**Moderate amounts of ascites were detected in case 1 after treatment with IL-11 by color doppler ultrasound: 2.1 cm in perihepatic region, 0.8 cm in perisplenic region, and 2.8 cm in the right lower abdomen**.

**Figure 3:**
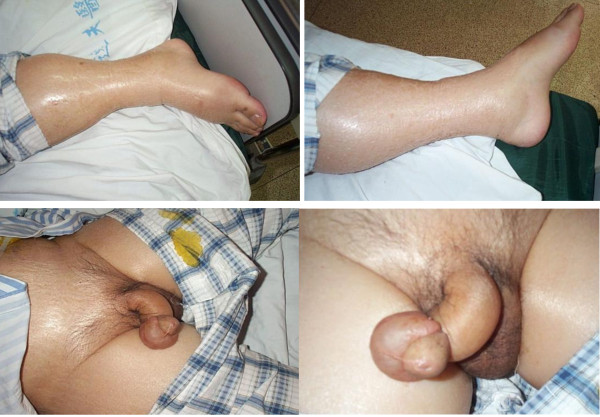
**Bilateral lower extremity edema, scrotum edema and penis edema were observed after treatment with IL-11 in case 1**.

Four days later, the patient felt fullness of the abdomen. His oral body temperature was 39.8°C, blood pressure was 102/61 mmHg, and pulse was 118 bpm. The breath sounds of the lower right lung were weak. Shifting dullness was detected in the abdomen. Laboratory values were as follows: leukocytes count 4.2 × 10^9^/L; hemoglobin 11.7 g/dL; hematocrit 34%; platelet count 46 × 10^9^/L. Piperacillin/Tazobactam was infused at a dose of 4.5 g q8 h for prophylaxis against infection. Indomethacin was administered against fever. However, the hypotension did not improve. At 21:00 on the same day, the patient complained of dyspnea and fatigue, his blood pressure was 86/52 mmHg, pulse was 120 bpm, and temperature was 40.5°C. The X-ray of his chest is shown in Figure 1. There were moderate amounts of ascites in the abdomen as shown by B ultrasound (Figure 2). As a consequence, fluid infusion was administered rapidly to this patient. However, the edema deteriorated dramatically and he developed oliguria, but pulmonary edema was not observed. The patient was transferred to the ICU department at 23:00, when his blood pressure was 76/49 mmHg, and pulse was 122 bpm. Fluid resuscitation and the administration of dopamine were initiated. On ICU day 2, the laboratory values were as follows: leukocytes count 7.0 × 10^9^/L; hemoglobin 7.5 g/dL; hematocrit 21.7%; platelet count 30 × 10^9^/L. Serum albumin had decreased to 1.8 g/dL. Therefore, packed red blood cells (pRBCs) 4.0 units with fresh frozen plasma (FFP) 400 ml were transfused into this patient at 02:00 and infusion of hydroxyethyl starch and albumin was started. On ICU day 3, serum albumin had increased to 2.3 g/dL. Over the following days, the hypovolemic shock and pleural effusion subsided gradually. However, on ICU day 4, edema of feet, the penis and scrotum appeared (Figure 3), and the patient was treated with albumin (10 g per day), spironolactone (40 mg per day), and hydrochlorothiazide (25 mg per day). The edema was alleviated gradually. After 19 days of treatment, the patient was discharged. On the day of discharge, the laboratory values were as follows: leukocytes count 5.2 × 10^9^/L; hemoglobin 11.3 g/dL; hematocrit 33.3%; platelet count 65 × 10^9^/L. Serum albumin had increased to 3.1 g/dL.

### Case 2

**Figure 4:**
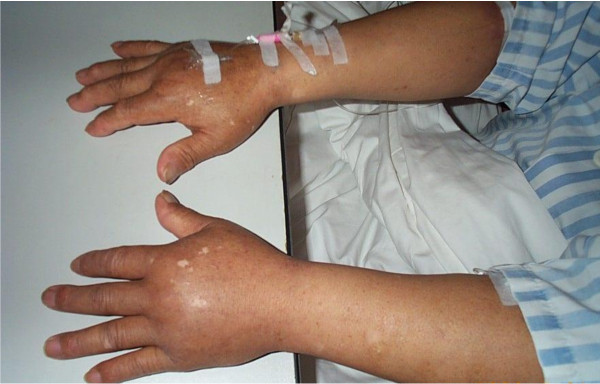
**After treatment with IL-11, bilateral edema of the hands were observed in case 2**.

On May 24^th ^2010, a 66-year-old man was admitted for primary liver cancer, with hypersplenia. On the day of admission, the blood pressure of the patient was 134/76 mmHg, heart rate was 75 bpm, respiratory rate was 19 bpm, and oral body temperature was 36.7°C. Laboratory values were as follows: leukocytes count 5.6 × 10^9^/L; hemoglobin 12.1 g/dL; hematocrit 34.8%; platelet count 63 × 10^9^/L; albumin 2.6 g/dL; AST 78 IU/L; and ALT 49 IU/L. After RFA, IL-11 was administered subcutaneously at a dose of 3 mg per day because of thrombocytopenia. Two days after the initiation of IL-11, the patient complained of dyspnea with bilateral edema of the feet. On physical examination, his temperature was 36.9°C, blood pressure was 93/59 mmHg, and pulse was 92 bpm. Fine rales could be heard by auscultation. Shifting dullness was detected in the abdomen. Edema of the hands was also observed (Figure 4). Laboratory values were as follows: leukocytes count 8.9 × 10^9^/L; hemoglobin 10.7 g/dL; hematocrit 30.6%; platelet count 51 × 10^9^/L; albumin 1.99 g/dL. Ascites puncture revealed a few red blood cells in the ascites and the level of albumin in the ascites was 1.3 g/dL. IL-11 was stopped and human albumin was infused at 10 g per day. Somatostatin was administered by intravenous push at a dose of 0.25 mg/hour. Platelets and FFP were also infused. On the 4^th ^day after the termination of IL-11, the serum creatinine (Cr) of the patient was 2.2 mg/dL (normal range 0.7-1.3 mg/dL), and fluid resuscitation was performed after consultation with the urology department. The 24-hour urine volume was 1500-2000 ml after the use of furosemide. Six days after the termination of IL-11, the edema of the feet and dyspnea were alleviated. Two weeks after the termination of IL-11, the patient was discharged. The laboratory values were as follows: leukocytes count 4.5 × 10^9^/L; hemoglobin 10.6 g/dL; hematocrit 30.2%; and platelet count 67 × 10^9^/L. Serum albumin had increased to 3.3 g/dL.

## Conclusions

IL-11 is a cytokine derived from stromal cells that has multiple effects on thrombocytopenia induced by chemotherapy or bone marrow transplantation (BMT). It was considered to be a safe and well tolerated agent for the treatment of thrombocytopenia after chemotherapy by several clinical trials [13-15]. In addition, IL-11 might be crucial in the acute management of certain types of hypersplenic thrombocytopenia in hepatic carcinoma [16]. Therefore, we choose IL-11 to platelet transfusion, which used to treat thrombocytopenia for preventing life-threatening bleeding episodes in patients with hepatic carcinoma from radiofrequency ablation. The most common side effects of IL-11 are arthralgias and myalgias, fatigue, nausea, and headache [13]. Mild edema and minor conjunctival bleeding are also associated with the administration of IL-11. The most severe side effects of IL-11 are tachycardia and hypotension, but these seldom occur [14]. To our knowledge, CLS due to the use of IL-11 has never been reported previously, particularly in liver cancer patients.

Herein, we describe two cases of liver cancer patients with anasarca, pleural effusion, and ascites. Clinical and laboratory findings were consistent with an acute episode of CLS. Treatment with hydroxyethyl starch, albumin, and diuretic led to a gradual improvement within 2 weeks. These findings should help to improve the understanding and treatment of this potentially deadly severe side effect of IL-11. Both patients had hypersplenia and elevated levels of ALT and AST, and it seemed likely that these findings were due to the side effects of a drug, such as IL-11, after RFA therapy. The first patient was treated with antibiotics after RFA for prophylaxis against infection because he had a high temperature. However, it became apparent from the subsequent pathogenesis that the symptoms described above were not due to infection, because the patient had hypoalbuminemia and the blood pressure continued to decrease. Some studies have demonstrated that IL-11 can be used in patients not only to promote platelet recovery but also to prevent life-threatening infections [17].

During the state of shock, hemoconcentration was not observed, but the hematocrit of the patient was decreased. As a consequence, hemorrhage should have been considered in this patient. However, in this patient, the level of hemoglobin and hematocrit increased to normal without the infusion of platelets and hemostasis. The patient did have pleural effusion, ascites and edema in both feet and the scrotum, which were all consistent with CLS, and thus it is likely that the main cause of the observed symptoms was CLS.

It was interesting that hemorrhage was also identified firstly in the second patient. According to some clinical trials, the proportion of hemorrhagic complications after RFA is approximately 1.5% [18]. Specific treatments, such as blood transfusion and drainage, are required in high-risk patients. However, in the second patient, the level of albumin in the ascites was higher than normal. Hypoalbuminemia and lower blood pressure occurred soon after the initiation of IL-11 and serum Cr was also increased because of hypovolemia. Therefore, the patient was not just a simple hemorrhage, and CLS needed to be considered. Finally, the symptoms of this patient were alleviated by combined treatment with hemostasis and fluid resuscitation, which confirmed that CLS was the main cause of the observed symptoms and hemorrhage might be a secondary reason. There is also the possibility that CLS can be induced by hemorrhage in patients with liver cancer.

The pathogenesis of CLS caused by IL-11 was not elucidated. Some studies have found that vascular endothelial growth factor (VEGF) is associated with CLS [19,20]. Furthermore, studies in animal models have shown that the synthesis of nitric oxide (NO) is strongly induced during CLS after cytokine treatment [21]. Another study found that isoforms of NO synthase (NOS), such as endothelial NOS, might play a major role in CLS [22]. Liver pathologies have a negative influence on numerous conditions, including those that affect the endothelial system [23]. In this report, both patients had chronic bleeding after RFA, which indicated that injury of the liver and hypersplenia might be a precipitating factor for CLS.

Further studies are necessary with the objective of collecting sufficient patients with CLS to observe the natural history of the disease and evaluate the efficacy of empiric treatments. These two cases should help to improve the understanding and treatment of the potentially deadly CLS that is related to IL-11 and has been recognized in this study.

## Consent statement

Written informed consent was obtained from the patient for publication of this case report and accompanying images. A copy of the written consent is available for review by the Editor-in-Chief of this journal.

## Competing interests

The authors declare that they have no competing interests.

## Authors' contributions

WKF carried out the patients' therapy, participated in analyzing the state of the two patients and drafted the manuscript. PHM participated in the design of the paper and performed the analysis. LHZ participated in design therapy project, estimate prognosis of the two patients. SLR and ZXY conceived of the study, and participated in its design and coordination. All authors read and approved the final manuscript.

## Pre-publication history

The pre-publication history for this paper can be accessed here:

http://www.biomedcentral.com/1471-2407/11/204/prepub
